# Cost-effectiveness analysis of typhoid vaccination in Lao PDR

**DOI:** 10.1186/s12889-023-17221-2

**Published:** 2023-11-17

**Authors:** Mick Soukavong, Nantasit Luangasanatip, Phetsavanh Chanthavilay, Yot Teerawattananon, Saudamini Vishwanath Dabak, Wirichada Pan-ngum, Tamalee Roberts, Elizabeth A Ashley, Mayfong Mayxay

**Affiliations:** 1 Faculty of Medicine, University of Health Sciences, Vientiane, Lao People’s Democratic Republic; 2 Mahidol Oxford Tropical Medicine Research Unit, Mahidol University, Bangkok, Thailand; 3 Unit for Health Evidence and Policy, Institute of Research and Education Development, University of Health Sciences, Vientiane, Lao People’s Democratic Republic; 4 Health Intervention and Technology Assessment Program, Ministry of Public Health, Nonthaburi, Thailand; 5 Saw Swee Hock School of Public Health, National University of Singapore, Singapore, Singapore; 6 Department of Tropical Hygiene, Faculty of Tropical Medicine, Mahidol University, Bangkok, Thailand; 7 Lao-Oxford-Mahosot Hospital-Wellcome Trust Research Unit, Microbiology Laboratory, Mahosot Hospital, Quai Fa Ngum, Lao People’s Democratic Republic, Vientiane, Laos; 8 Centre for Tropical Medicine and Global Health, Nuffield Department of Clinical Medicine, University of Oxford, Oxford, UK

**Keywords:** Typhoid, Enteric Fever, Conjugate vaccine, Lao PDR, Cost-effectiveness

## Abstract

**Background:**

Typhoid vaccination has been shown to be an effective intervention to prevent enteric fever and is under consideration for inclusion in the national immunization program in Lao PDR.

**Methods:**

A cost-utility analysis was performed using an age-structured static decision tree model to estimate the costs and health outcomes of introducing TCV. Vaccination strategies combined with five delivery approaches in different age groups compared to no vaccination were considered from the societal perspective, using the Gavi price of 1.5 USD per dose. The vaccination program was considered to be cost-effective if the incremental cost-effectiveness ratio was less than a threshold of 1 GDP per capita for Lao PDR, equivalent to USD 2,535 in 2020.

**Results:**

In the model, we estimated 172.2 cases of enteric fever, with 1.3 deaths and a total treatment cost of USD 7,244, based on a birth cohort of 164,662 births without TCV vaccination that was followed over their lifetime. To implement a TCV vaccination program over the lifetime horizon, the estimated cost of the vaccine and administration costs would be between USD 470,934 and USD 919,186. Implementation of the TCV vaccination program would prevent between 14 and 106 cases and 0.1 to 0.8 deaths. None of the vaccination programs appeared to be cost-effective.

**Conclusions:**

Inclusion of TCV in the national vaccination program in Lao PDR would only be cost-effective if the true typhoid incidence is 25-times higher than our current estimate.

**Supplementary Information:**

The online version contains supplementary material available at 10.1186/s12889-023-17221-2.

## Background

Typhoid fever is an exclusively human, enterically transmitted systemic disease caused by infection with the bacterium *Salmonella enterica* serovar Typhi (*S.*Typhi). *S.*Typhi is responsible for an estimated 11–20 million infections and 128,000–161,000 deaths annually in resource-poor countries, which often lack access to clean water and have poor sanitary facilities [1, 2]. Children are disproportionately affected by typhoid fever, with peak incidence in the 5–15 years age group [3]. The standard threshold to define high typhoid incidence is ≥ 100 cases per 100,000 population per year [4].

Lao PDR (or Laos) is a lower-middle income country in southeast Asia which shares borders with Thailand, Vietnam, Cambodia, Myanmar and China, and has a population of 7.2 million. Annual expenditure on healthcare is around 2.6% of its Gross Domestic Product (GDP). The true incidence of typhoid fever is not well documented due to a lack of diagnostic microbiology in many parts of the country. In an analysis of eighteen years of blood culture data from Mahosot Hospital, a primary to tertiary care hospital in Vientiane, published in 2020, a total of 913 (1.5%) of 60,384 blood cultures were positive for *S.* Typhi. Multi-drug resistance (resistance to chloramphenicol, ampicillin, and cotrimoxazole) was detected in 59 (6.5%) of the 898 isolates with antibiotic susceptibility results available [5]. Minimum crude estimates of incidence for hospitalized typhoid cases were calculated, which were generally below one case per 100,000 people/year, and highest among 6 to 20 year olds. A separate analysis of data from 2015 to 2017 estimated an incidence of 4.7 per 100,000 persons in the hospital’s catchment area (Vientiane capital), factoring in healthcare utilization and blood culture sensitivity [6].

At present, there are three typhoid vaccines available: (i) the oral live attenuated vaccine (Ty21a), (ii) the parenteral unconjugated Vi polysaccharide vaccine (ViPS), and (iii) the parenteral typhoid conjugate vaccine (TCV). ViPS and Ty21a vaccines can be administered to patients above the age of two and six years, respectively, while TCV is administered to infants aged 6 months and above [3]. TCV efficacy was estimated at 87.1% [95% CI: 47.2–96.9%] in human challenge studies in adults [7]. Based on a World Health Organization (WHO) vaccine position paper, TCV is preferred in all age groups in view of its improved immunogenic properties; suitability for use in younger children; and expected longer duration of protection (> 3 years) compared to other vaccines (3–7 years for Ty21a, 2 years for ViPS) [8]. WHO recommends to administer TCV at 9 months or in the second year of life and catch-up TCV again up to 15 years of age, if supported by local epidemiological data [3]. Despite the recommendation by WHO in 2008 that typhoid vaccination should be considered for the control of endemic disease and outbreaks, programmatic use remains limited.

Current immunization coverage for fully immunized children aged 12 to 23 months in Laos is only 48%, which falls significantly below the government’s target of 90%. Additionally, there is a lack of equity in coverage, with higher levels of immunization rates found among wealthier and urban families [9]. Low uptake of facility-based birthing and high dropout rates are two primary factors contributing to low immunization coverage in rural areas. Implementing school-based vaccination programs and conducting vaccination record checks at school can potentially enhance vaccination coverage among school-aged children [10]. A combination of community and school-based strategies is one approach that may reach both children and adults in high-incidence settings where all ages are at risk.

In Laos, typhoid vaccine is currently not part of the national immunization program but introduction is being considered. Gavi, the Vaccine Alliance, has been supporting the Lao immunization program since 2002 with the expectation that the government will transition to a self-sustaining program by 2026. Before considering adding another vaccine to the Expanded Program on Immunization (EPI) cost-effectiveness needs to be considered.

Fewer than 20 economic evaluations of typhoid vaccines have been published. All of these studies evaluated “vaccination” as the intervention, and compared it to a “no vaccination” scenario and the majority reported the typhoid vaccine to be a cost-effective intervention. A mathematical modelling study by Pitzer et al. predicted that TCV administered to children aged 9 months prevents more cases of typhoid compared to the live oral vaccine [11]. Common factors which influenced cost-effectiveness of the vaccine included incidence, case fatality rate, vaccine cost, duration of protection, and vaccine effectiveness. Although herd immunity and sanitation and hygiene are considered to be important benefits of the vaccination, accounting for them is challenging, and very few studies have done so.

One study in 2019 determined the cost-effectiveness of TCV compared to no vaccine in 54 Gavi-eligible countries, including Laos. This study compared four strategies: (i) no vaccination; (ii) routine immunization at 9 months; (iii) routine immunization at 9 months with catch-up campaigns to either age 5 years and (iv) routine immunization at 9 months with catch-up campaigns to 15 years, and highlighted that typhoid vaccination was likely to be the preferred intervention if the Lao government has a willingness-to-pay (WTP) of at least 300 USD per disability-adjusted life year (DALY) averted. Specifically, with a WTP of 1 GDP per capita in Laos, there was a 92% chance that the intervention would be cost-effective. However, this study assumed that the median [95% credible interval] annual typhoid incidence in Laos was 614 [362 to 871] per 100,000 person-years which is many times higher than current evidence suggests [12].

This study aims to assess the cost-effectiveness of TCV in Laos using local data and considering a variety of delivery approaches for TCV to inform immunization policy in the country.

## Methods

A model-based economic evaluation was performed to estimate the costs and health outcomes of introducing TCV typhoid vaccine. A cost-utility analysis was conducted using Quality Adjusted Life Years (QALYs). Recent data has shown higher incidence among the population aged 6–20 years and highest in young adults aged 16–20 years, [5] therefore we assessed five delivery approaches: (i) school-based (SchB), (ii) community-based (ComB), starting at 9 months of age, (iii) community-based with a catch-up campaign at age 15 years (ComB + 15y), (iv) community-based with a catch-up campaign at age 20 years (ComB + 20y) and (v) school-based with a catch-up campaign at age 21 years (SchB + 21y) (see additional file for summary of schedules). These were compared to a scenario of no vaccination in Laos from the societal perspective. The incremental cost-effectiveness ratio (ICER) for each of the vaccination strategies was estimated. A life-time horizon with a 3% discounting rate for both cost and outcomes was used. We used Microsoft Excel software to run and analyze the model outputs.

### Modeling approach

An age-structured static decision tree model with typhoid infections was constructed. A fixed number of newborns in an initial cohort was estimated from the World Bank birth rate data and Lao official population data from the Laos Statistics Bureau [13] at the beginning of the model simulation. For each one year time step, the number of survivors in each age group was estimated using age-specific mortality data from the Lao Statistics Bureau [13]. The number of typhoid infections per year was calculated based on the age-specific incidence of typhoid and death from Roberts T et al. 2020 [see supplementary material], using a multiplier of 2 which is derived from an estimated sensitivity of blood culture for *S*.Typhi of 50% [4]. The natural history of typhoid disease was incorporated in to the probabilistic branches of the decision tree in order to assess costs and health outcomes of different national vaccination programs in Laos (see supplementary material). Given the low prevalence of multi-drug resistance in Laos we did not consider this in the model. Typhoid fever cases were classified into two health states; uncomplicated and with complications. Uncomplicated typhoid is characterized by prolonged fever, disturbances of bowel function, headache, cough, malaise and anorexia [14]. Typhoid with complications describes patients who develop intestinal perforation, peritonitis, altered mental status, delirium, coma and haemorrhage [14]. Typhoid cases with and without complications have different risks of death due to difference in severity. The model was run for the life time horizon of 100 years. All parameter inputs are shown in Table 1.

**Table 1:** The parameters input in the model

Parameter	Values	(95% CI)	Source
**Epidemiology Data**			
Population size (Lao PDR)	Population by age group		[13]
Death General Population	Death General Population by age group		WHO (2019)
Surveillance data for reported Typhoid cases	Typhoid cases by age group1		[5]
Number of births per year	164,662.39		[30]
**Risk of severity-typhoid**			
Risk of typhoid without complications	0.737	68–79	[31]
Risk of typhoid with complications	0.263	21–32	[31]
Risk of death due to Typhoid without complications	0.002	0–0.007	[31]
Risk of death due to Typhoid with complications	0.024	0.006–0.015	[31]
**Intervention**			
Vaccine efficacy TCV	87.50%	80–95%	[7, 32]
Vaccine coverage	> 6 months of age		[7, 32]
Vaccine mean duration	15 years	10–20 years	[7, 32]
**Vaccination program cost**			
Vaccine price	1.5 USD/dose		[15]
School-based cost per dose	3.99 (adjusted with CPI 2013–2020 (3.33 USD) 2013		[16]
Community-based routine	1.36 USD	0.44–3.32	[17]
**Cost**			
Cost of typhoid without complications	14 USD		Expert Opinion
Cost of typhoid with complications	155.19 USD		Local data collection
Direct non-medical cost without complications	0.7 USD		Local data collection
Direct non-medical cost with complications	29.64 USD		Local data collection
Indirect cost without complications	25 USD		PwC tax summaries (2020)
Indirect cost with complications	50 USD		PwC tax summaries (2020)
Utility of typhoid with complications	0.59		
Utility of typhoid without complications	0.94	0.89–0.99	[21]
Days of typhoid illness (with complications)	7		[21]
Days of typhoid illness (without complications)	3		[21]
Discount rate for cost	0.03		
Discount rate for outcome	0.03		
Parameter	Values	(95% CI)	Source

^1^ Typhoid incidence data by age group in supplementary material

### Costs, utility and analysis

#### Cost of vaccine

Cost of vaccination included both the price of vaccine as well as its service delivery cost. A price per dose of USD 1.5 based on the Gavi price was assumed in this analysis [15]. We assumed that the TCV will be delivered through existing health facilities, but not concurrently with other vaccines or other interventions, and used estimates of a school-based scenario cost per dose at USD 3.99 (adjusted with CPI 2013–2020) and community-based routine cost per dose at USD1.36 based on other studies in Laos [16, 17].

### Direct medical costs data collection

Only direct medical costs associated with typhoid cases were considered. Data were extracted from accessible medical records of patients previously hospitalized in Mahosot Hospital, Vientiane, with a laboratory confirmed diagnosis of enteric fever. Some authors had access to information that could identify individual participants since the blood cultures were processed in the hospital laboratory as part of their routine care. The calculation of direct medical costs was performed by multiplying the quantities of resources used by their unit costs. Financial resources comprised three elements: (1) resources consumed, (2) frequency of resources used and (3) quantifying the value of these resources. Resources were broken down into five categories: (1) diagnostic investigation (laboratory tests, radiology etc.), (2) treatment, e.g. medications, fluids etc., including operation costs if applicable, (3) supplies and disposable equipment, (4) hospital services (daily bed costs, etc.) and (5) personnel (time spent attending patients). The personnel costs were considered separately from hospital services because salaries and incentives are paid by the government. Hospital service resources are partially a representation of overhead costs, which include hospital-bed days and outpatient consultation fees.

Direct non-medical costs and indirect costs covering transportation, additional food, and caregiver costs (children) and productivity lost due to the caregivers taking their child to visit healthcare facilities or the patients themselves (in adult cases) were obtained from literature review.

Health outcomes were measured as number of cases, deaths, and QALYs. We derived estimates of health-related quality of life of two health states; typhoid with and without complications, which were taken from literature review due to the limited number of annual typhoid cases on which to directly perform utility assessment in the Lao population. As such, QALY losses were calculated using a utility decrement for patients with typhoid with and without complications. QALY losses due to mortality were also calculated using the conditional life expectancy at the corresponding age.

The results were expressed as the incremental cost-effectiveness ratio (ICER) in USD per QALY gained with the willingness-to-pay (WTP) threshold at 1 GDP per capita for Lao PDR which was equivalent to USD 2,535 in 2020 [18].

### Sensitivity analysis

One-way sensitivity analysis was performed by changing one parameter by their range of values at a time to see the impact on ICER. The parameters explored included incidence rate, vaccine duration of protection, vaccine efficacy, vaccine cost and vaccine coverage. Incidence was allowed to vary by up to seven times based on recent data from a study of inpatients and outpatients of all ages presenting with febrile illness at a hospital in Vientiane province in Laos [19]. Probabilistic sensitivity analysis (PSA) using a Monte Carlo simulation was also carried out to assess uncertainty of all parameter inputs. For each parameter, one value was drawn from the assigned distribution simultaneously to calculate cost and effectiveness. This process was then repeated 1,000 times and results were presented as cost-effectiveness acceptability curves (CEAC).

### Budget impact analysis

The financial impact of a national typhoid vaccine implementation with a 5-year timeframe from the government perspective was estimated. All relevant information including cost of vaccine, cost of administration and cost of treatment with different scenarios of vaccine uptake were combined to estimate the budget impact.

We followed the Consolidated Health Economic Evaluation Reporting Standards 2022 (CHEERS2022) Statement: Updated Reporting Guidance for Health Economic Evaluations (see supplementary material).

## Results

A closed cohort of 164,662 newborns was simulated in the model to estimate lifetime burden of Typhoid fever. In our base case scenario, i.e. without a TCV vaccination program, we estimated the total number of typhoid fever cases as 172.2, with 1.3 deaths and the total cost of treatment of USD 7,244 over the maximum life-time horizon of 100 years. These estimates were equivalent to the estimated incidence of 2.17/100,000 population, which is consistent with the reported incidence with a multiplier of 2 suggested previously [20]. To implement a TCV vaccination program, the cost of the vaccine and administration were calculated to be USD 700,272, USD 470,934, USD 740,371, USD 699,475 and USD 919,186 for school-based (SchB), community-based (ComB), community-based with catch-up campaigns at age 15 years (ComB + 15y), community-based with catch-up campaigns at age 20 years (ComB + 20y) and school-based with catch-up campaigns at age 21 years (SchB + 21y), respectively. The corresponding costs of treatment for these programs would be USD 4,298, USD 6,291, USD 2,453 USD 3,892 and USD 2,090. Implementation of the TCV vaccination program would prevent between 14 and 106 cases and 0.1 to 0.8 deaths during the program’s implementation. Additionally, the incremental QALYs would be 14.8, 5.3, 23.1, 15.3, and 24.0 for SchB, ComB, ComB + 15y, ComB + 20y, and SchB + 21y, respectively Table 2.

**Table 2:** Health benefits and economic impact results of TCV vaccines

Vaccine option	Case of Typhoid	Death of Typhoid	Cost of vaccine	Cost of vaccine administration	Cost of treatment	Case avoided	Death avoided
Baseline: No vaccine	172.2	1.3			7,243.71		
School-based	120.4	0.9	191,331.05	508,940.58	4,297.97	51.82	0.4
Community-based	158.3	1.2	246,993.59	223,940.85	6,290.53	13.90	0.1
Community-based + 1 time catch up at 15 years old	80.2	0.6	388,306.34	352,064.41	2,452.55	92.04	0.7
Community-based + 1 time catch up at 20 years old	100.5	0.8	366,051.25	333,424.11	3,892	71.73	0.6
School-based + 1 time catch up at 21 years old	66.1	0.5	306,146.31	613,039.75	2,090.37	106.10	0.83

The ICER of the five vaccination programs compared to a no vaccination scenario were USD 47,069.28, USD 89,078.24, USD 31,820.94, USD 45,444.21 and USD 38,162.79 per QALY gained for the school-based, community-based, community-based with catch-up campaigns at age 15 years, community-based with catch-up campaigns at age 20 years and school-based with catch-up campaigns at age 21 years, respectively, Table 3.

**Table 3:** Incremental cost-effectiveness ratio results of TCV vaccine compared to no vaccine

Vaccine option	Total cost (USD, 2021)	QALYs loss	Incremental cost	Incremental QALYs	ICER
Baseline: No vaccine	7,243.71	31.8			
School-based	704,569.60	16.9	697,325.89	14.8	47,069.28
Community-based	477,224.97	26.5	469,981.26	5.3	89,078.24
Community-based + 1 time catch up at 15 years old	742,823.30	8.6	735,579.59	23.1	31,820.94
Community-based + 1 time catch up at 20 years old	703,367.21	16.4	696,123.50	15.3	45,444.21
School-based + 1 time catch up at 21 years old	921,276.43	7.80	914,032.73	23.95	38,162.79

**Fig. 1:**
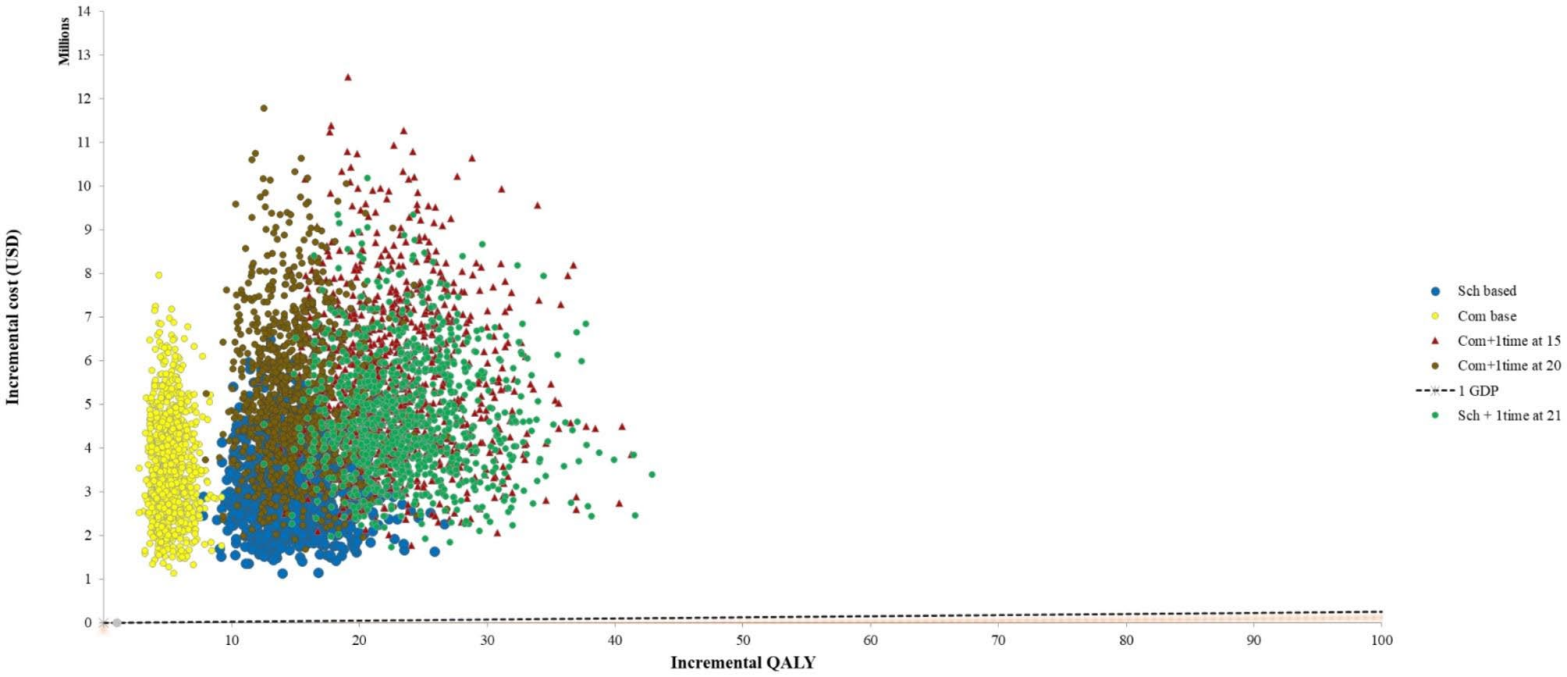
Probabilistic sensitivity analysis (PSA) results showing all 1,000 iterations of each vaccine option compared with no vaccine (base case). (Exchange rate, USD 1 = 10,000 Lao Kip)

At a threshold of one times the GDP per capita (USD 2,534.9), none of the vaccination programs were cost-effective as the ICER exceeded this threshold from the societal perspective. A higher incidence (starting with a multiplier of 25 times the incidence) would make certain vaccine strategies cost-effective using the Gavi price of 1.5 USD per dose. Conversely, using the market price of TCV (USD 20.17 per dose), a higher incidence, starting from a multiplier of 150-times, would also result in some vaccine strategies becoming cost-effective. The PSA using Monte Carlo simulation repeating the analysis with one thousand runs and sampling from a set of parameter probability distributions with a threshold of 1 GDP per capita is shown in Fig. 1.

**Fig. 2:**
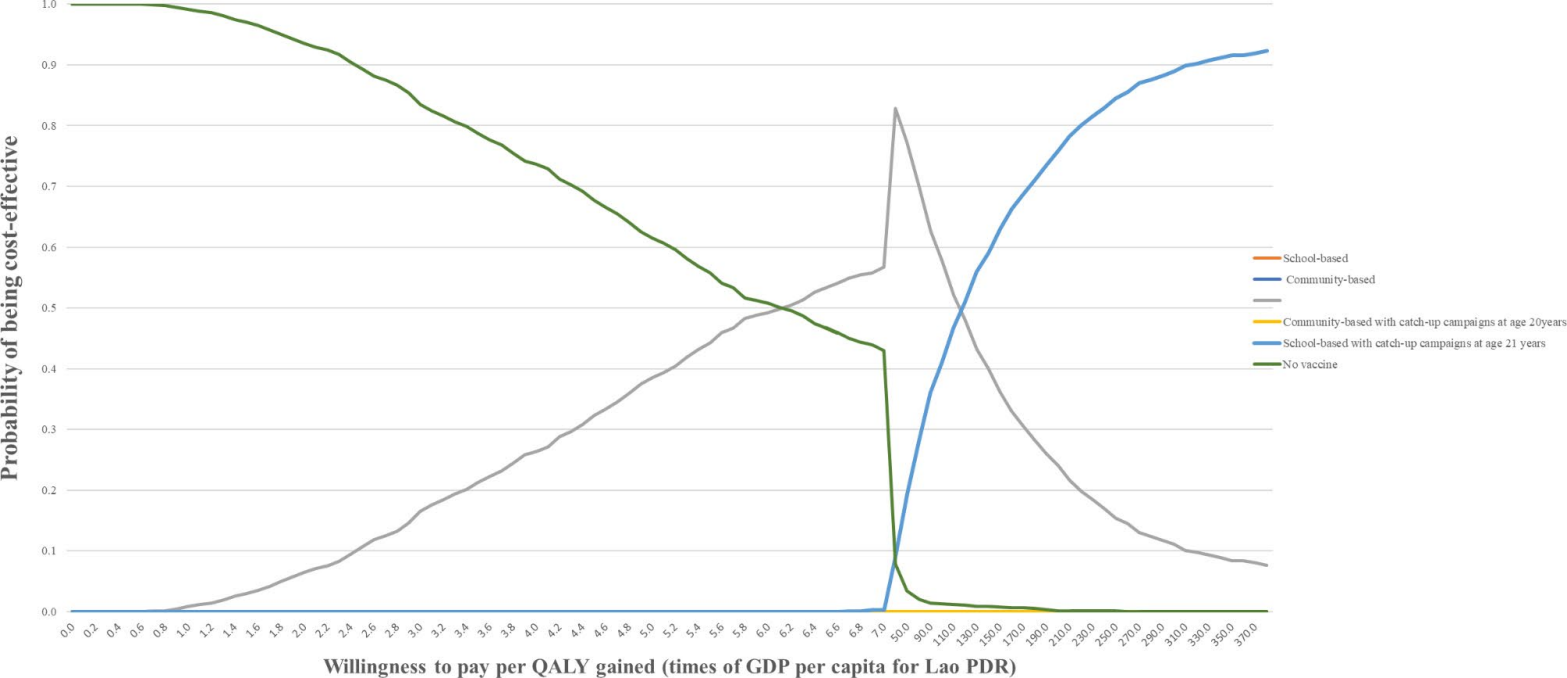
Cost-effectiveness acceptability curve

The CEAC shows the result of multivariate probabilistic sensitivity analysis based on 1,000 Monte Carlo simulations. The probability of favouring each option is dependent on the level of willingness to pay. At WTP threshold of 1 GDP no vaccine (99.1%) was the best option, If the WTP threshold is set at 7 times one GDP per capita (USD 17,744), ComB + 15y had the highest chance (56.7%) of being cost-effective followed by no vaccine (43%) and SchB + 21y (0.3%) (Fig. 2).

Based on the budget impact analysis conducted, implementing universal typhoid vaccination by any approach in Laos for five years would cost between USD 0.4 to 1.3 million per year, with the vaccine alone costing USD 0.2 to 0.5 million per year (Supplementary material).

## Discussion

The results of our cost-effectiveness analysis indicate that typhoid vaccination would not be cost-effective in Laos. This is in contrast to previous studies in other settings that have shown that typhoid vaccination is cost-effective [12, 21]. The main factor contributing to lack of cost-effectiveness was low disease incidence. The annual typhoid incidence estimated by our model was 2.17/100,000 population, based on data of laboratory-confirmed cases in Laos, and incorporating a commonly used multiplier of two to take into account the insensitivity of blood culture for detecting *S*.Typhi. While this is likely an underestimation of the true incidence since the data were from passive surveillance of patients presented to selected provincial and central hospitals, another study in Vientiane which used health care utilization to derive estimates of incidence only estimated an incidence of 4.7/100, 000. This is well below the estimate of 614 per 100,000 person-years used in another recent study that predicted introduction of TCV would be cost-effective [12]. This shows that the approach to estimating typhoid incidence is not standardized with a variety of different multipliers being used [22].

A low incidence of typhoid has also been reported in two neighboring countries. In Thailand, typhoid incidence decreased from 8.6 cases per 100,000 in 2003 to 3 cases per 100,000 in 2014 [23]. A similar reduction has been reported in Vietnam, even though rates of multi-drug resistance were previously high. These reductions have been attributed to improvements in water availability and quality, hygiene and sanitary facilities. Both countries initiated several vaccination campaigns, starting in the 1970s in Thailand and 1997 in Vietnam, which may also have contributed to the decrease in incidence [23, 24]. Currently typhoid vaccine is not included in the EPI in Thailand and is only recommended for use in children (3–10 years old) in high-risk areas in Vietnam. In contrast, a study in 2018 in Yangon, Myanmar, which also borders Laos, estimated the annual incidence of typhoid at 391 per 100,000 population [25].

Using the Gavi price of TCV (USD 1.5) some vaccine strategies would be cost effective if the true incidence is 25 times higher than our base estimate. If we considered the market price of TCV instead, some vaccine strategies would be cost effective only if the incidence of typhoid fever was 150 times higher.

Gavi has been supporting immunization in Laos for more than 20 years but is planning to withdraw over the next three years. There are four programmatic challenges that transitioning countries are confronting: decision-making; political commitment and financial sustainability; equitable delivery of vaccines; and access to timely and affordable supply [26]. Financing the immunization programme is expected to be very challenging. Following the COVID-19 pandemic, Laos has experienced a major economic downturn due to loss of tourism, trade and investment, rising consumer goods prices, declining remittance and weak exchange rates.

Our study has limitations. We did not assume any herd immunity or indirect effects of vaccination. Due to the fact that the size of our target groups was relatively small compared to the size of the population in Laos, it could be expected that the herd immunity effects would be quite marginal and would not substantially affect our conclusions. We also did not consider vaccine as a strategy to avert antimicrobial drug resistance and associated costs, since there is very little multi-drug resistant *S*.Typhi in Laos currently; however this may change in the future. We could not assess a strategy of regional implementation of a vaccination program, e.g. targeting high burden areas, due to a lack of incidence data from the majority of provinces. Since there is no specific cost-effectiveness threshold for Laos, we used a common cost-effectiveness threshold (per capita GDP) as a proxy. This threshold is controversial, does not take into account health opportunity costs, and the results have profound consequences for resource allocation [27–29]. Additionally, this study did not consider the other two types of typhoid vaccine. As such, we cannot draw any conclusions regarding vaccine choice in this analysis. Finally, we should stress that the study findings do not reflect the financial affordability by the Lao government. More accurate data on the burden of this disease in Lao PDR would provide important information for decision makers to confirm these findings.

## Conclusion

Based on the current incidence and treatment costs of typhoid fever in Laos, inclusion of TCV vaccination in the national vaccination program in Laos would not be cost effective. The vaccine could become cost-effective if the typhoid incidence is 25-times higher than our current estimate or if the market vaccine price was used in conjunction with at least a 150-times increase in incidence of typhoid. The study calls for further research in this area in Laos to inform policy.

### Electronic supplementary material

Below is the link to the electronic supplementary material.

Supplementary Material 1

## Data Availability

Source data are provided in the supplementary material.
