# Supplementary material for: Cost-effectiveness analysis of typhoid vaccination in Lao PDR
Source: BMC Public Health. 2023 Nov 17;23:2270. doi: 10.1186/s12889-023-17221-2 (PMC10656839; doi:10.1186/s12889-023-17221-2)
Supplement: Supplementary file 1 — Supplementary Material 1 [file 12889_2023_17221_MOESM1_ESM.docx]

Additional File

**Vaccine schedules under consideration**

| **Vaccine** | **Age of routine vaccination** |
| --- | --- |
| School-based routine vaccination | |
| TCV | 6 years old |
| Community-based routine vaccination | |
| TCV | 9 months |
| Community-based routine vaccination + One-time catch-up campaign | |
| TCV | 9 months + (one-time catch up at 15 years old) |
| TCV | 9 months + (one-time catch up at 20 years old) |
| School-based routine vaccination + One-time catch-up campaign | |
| TCV | 6 years old + (one-time catch up at 21 years old) |

**S1 Table. Budget impact analysis**

| **Vaccine option** | **Year 1** | | | |
| --- | --- | --- | --- | --- |
|  | **Cost of vaccine** | **Cost of vaccine administration** | **Cost of treatment** | **Total Cost** |
| No vaccine |  |  | 10,193.45 | 10,193.45 |
| School Base | 228,459.27 | 607,701.67 | 7,126.80 | 843,287.74 |
| Community Base | 246,993.59 | 223,940.85 | 9,370.69 | 480,305.13 |
| Community Base + 1 time catch up at 15 year old | 467,154.25 | 423,553.18 | 4,746.38 | 895,453.81 |
| Community Base + 1 time catch up at 20 year old | 462,024.97 | 421,679.80 | 5,948.23 | 889,652.99 |
| School Base + 1 time catch up at 21 year old | 442,049.49 | 801,356.79 | 3,914.21 | 1,247,320.49 |
| **Vaccine option** | **Year 2** | | | |
|  | **Cost of vaccine** | **Cost of vaccine administration** | **Cost of treatment** | **Total Cost** |
| No vaccine |  |  | 9,489.89 | 9,489.89 |
| School Base | 212,690.75 | 565,757.39 | 6,634.90 | 785,083.03 |
| Community Base | 229,945.80 | 208,484.19 | 8,723.91 | 447,153.90 |
| Community Base + 1 time catch up at 15 year old | 434,910.71 | 394,319.05 | 4,418.78 | 833,648.54 |
| Community Base + 1 time catch up at 20 year old | 430,135.46 | 392,574.97 | 5,537.67 | 828,248.10 |
| School Base + 1 time catch up at 21 year old | 411,538.71 | 746,046.21 | 3,644.05 | 1,161,228.97 |
| **Vaccine option** | **Year 3** | | | |
|  | **Cost of vaccine** | **Cost of vaccine administration** | **Cost of treatment** | **Total Cost** |
| No vaccine |  |  | 9,271.60 | 9,271.60 |
| School Base | 207,798.43 | 552,743.82 | 6,482.28 | 767,024.52 |
| Community Base | 224,656.58 | 203,688.63 | 8,523.24 | 436,868.45 |
| Community Base + 1 time catch up at 15 year old | 424,906.88 | 385,248.91 | 4,317.14 | 814,472.93 |
| Community Base + 1 time catch up at 20 year old | 420,241.47 | 383,544.94 | 5,410.30 | 809,196.71 |
| School Base + 1 time catch up at 21 year old | 402,072.48 | 728,885.63 | 3,560.23 | 1,134,518.34 |
| **Vaccine option** | **Year 4** | | | |
|  | **Cost of vaccine** | **Cost of vaccine administration** | **Cost of treatment** | **Total Cost** |
| No vaccine |  |  | 9,075.54 | 9,075.54 |
| School Base | 203,404.18 | 541,055.13 | 6,345.20 | 750,804.52 |
| Community Base | 219,905.84 | 199,381.30 | 8,343.01 | 427,630.14 |
| Community Base + 1 time catch up at 15 year old | 415,921.52 | 377,102.18 | 4,225.85 | 797,249.55 |
| Community Base + 1 time catch up at 20 year old | 411,354.77 | 375,434.25 | 5,295.89 | 792,084.90 |
| School Base + 1 time catch up at 21 year old | 393,569.99 | 713,472.13 | 3,484.94 | 1,110,527.07 |
| **Vaccine option** | **Year 5** | | | |
|  | **Cost of vaccine** | **Cost of vaccine administration** | **Cost of treatment** | **Total Cost** |
| No vaccine |  |  | 8,859.76 | 8,859.76 |
| School Base | 198,567.95 | 528,190.74 | 6,194.33 | 732,953.02 |
| Community Base | 214,677.25 | 194,640.71 | 8,144.64 | 417,462.60 |
| Community Base + 1 time catch up at 15 year old | 406,032.37 | 368,136.01 | 4,125.37 | 778,293.75 |
| Community Base + 1 time catch up at 20 year old | 401,574.19 | 366,507.74 | 5,169.97 | 773,251.90 |
| School Base + 1 time catch up at 21 year old | 384,212.28 | 696,508.27 | 3,402.08 | 1,084,122.63 |

**S2 Table Age- specific incidence estimates of typhoid fever used in the model**

**Supplementary Fig 1*:* Cohort Model**


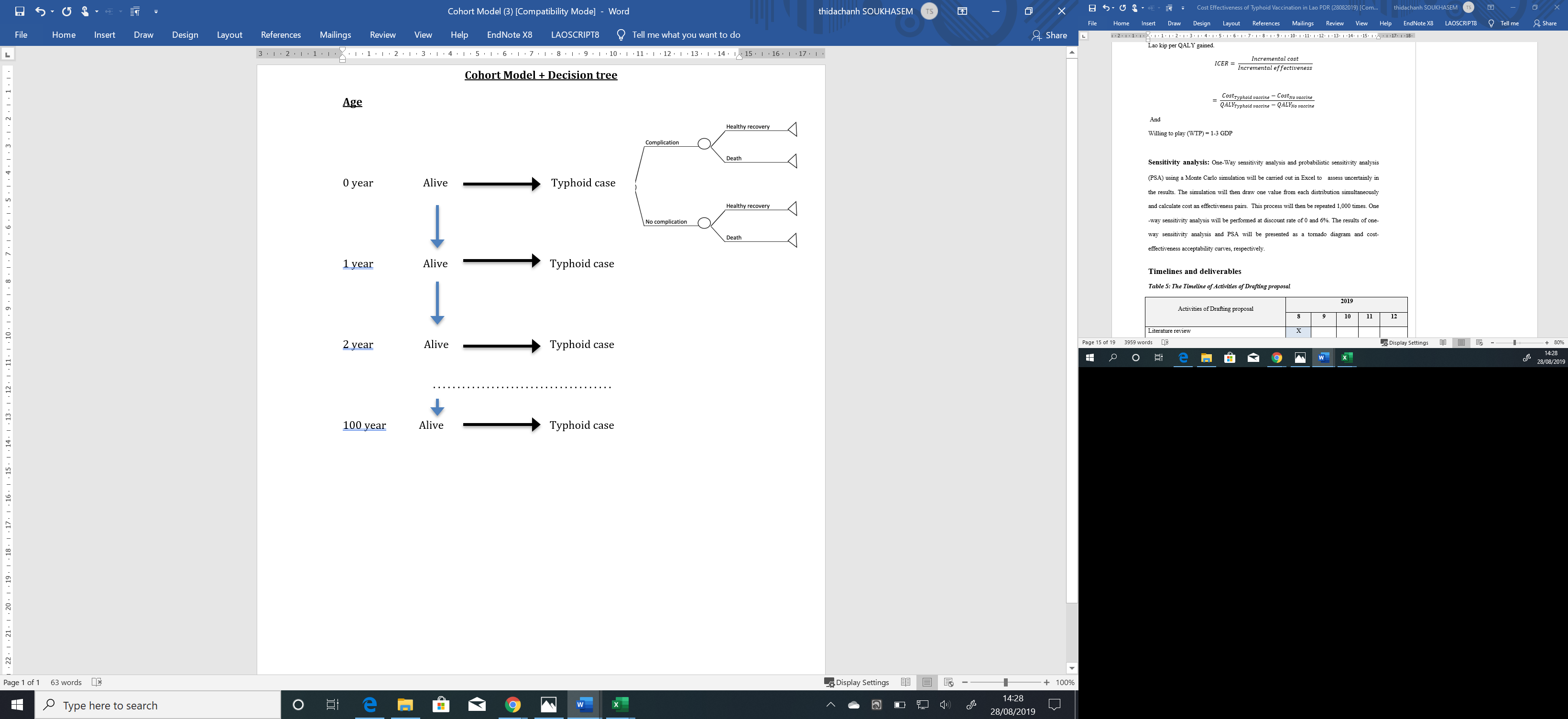


**Supplementary Fig 2: Decision Tree Model**


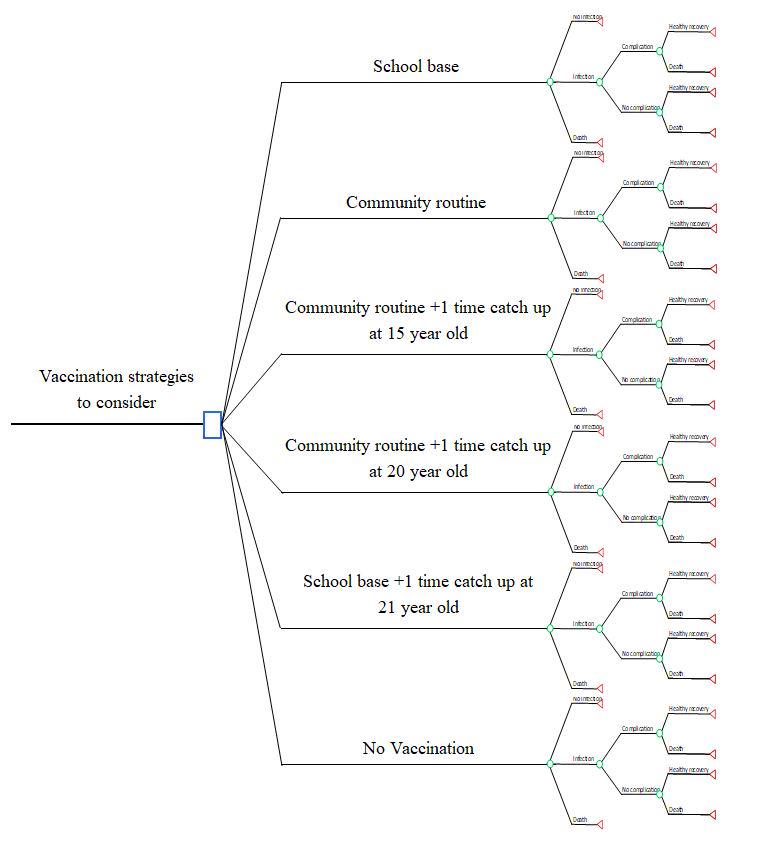

**CHEERS Checklist**

| **Topic** | **No.** | **Item** | **Location where item is reported** |
| --- | --- | --- | --- |
| **Title** |  |  |  |
|  | 1 | Identify the study as an economic evaluation and specify the interventions being compared. | Title |
| **Abstract** |  |  |  |
|  | 2 | Provide a structured summary that highlights context, key methods, results, and alternative analyses. | Abstract |
| **Introduction** |  |  |  |
| **Background and objectives** | 3 | Give the context for the study, the study question, and its practical relevance for decision making in policy or practice. | Introduction |
| **Methods** |  |  |  |
| **Health economic analysis plan** | 4 | Indicate whether a health economic analysis plan was developed and where available. | Methodology, First Paragraph |
| **Study population** | 5 | Describe characteristics of the study population (such as age range, demographics, socioeconomic, or clinical characteristics). | Methodology, Second Paragraph |
| **Setting and location** | 6 | Provide relevant contextual information that may influence findings. | Methodology, Second Paragraph |
| **Comparators** | 7 | Describe the interventions or strategies being compared and why chosen. | Methodology, Second Paragraph |
| **Perspective** | 8 | State the perspective(s) adopted by the study and why chosen. | Methodology, Third Paragraph |
| **Time horizon** | 9 | State the time horizon for the study and why appropriate. | Methodology, Sixth Paragraph |
| **Discount rate** | 10 | Report the discount rate(s) and reason chosen. | Methodology, Sixth Paragraph |
| **Selection of outcomes** | 11 | Describe what outcomes were used as the measure(s) of benefit(s) and harm(s). | Methodology, Fifth Paragraph |
| **Measurement of outcomes** | 12 | Describe how outcomes used to capture benefit(s) and harm(s) were measured. | Methodology, Direct Medical Costs Data Collection |
| **Valuation of outcomes** | 13 | Describe the population and methods used to measure and value outcomes. | Methodology, Direct Medical Costs Data Collection |
| **Measurement and valuation of resources and costs** | 14 | Describe how costs were valued. | Methodology, Costs, Utility and Analysis |
| **Currency, price date, and conversion** | 15 | Report the dates of the estimated resource quantities and unit costs, plus the currency and year of conversion. | Methodology, Direct Medical Costs Data Collection, Last Paragraph |
| **Rationale and description of model** | 16 | If modelling is used, describe in detail and why used. Report if the model is publicly available and where it can be accessed. | Methodology, Modelling approach, First and Second Paragraph |
| **Analytics and assumptions** | 17 | Describe any methods for analysing or statistically transforming data, any extrapolation methods, and approaches for validating any model used. | Methodology, Modelling approach, Third Paragraph |
| **Characterising heterogeneity** | 18 | Describe any methods used for estimating how the results of the study vary for subgroups. | Methodology, Sensitivity analysis |
| **Characterising distributional effects** | 19 | Describe how impacts are distributed across different individuals or adjustments made to reflect priority populations. | Methodology, Sensitivity analysis |
| **Characterising uncertainty** | 20 | Describe methods to characterise any sources of uncertainty in the analysis. | Methodology, Sensitivity analysis, Last paragraph |
| **Approach to engagement with patients and others affected by the study** | 21 | Describe any approaches to engage patients or service recipients, the general public, communities, or stakeholders (such as clinicians or payers) in the design of the study. | Not applicable |
| **Results** |  |  |  |
| **Study parameters** | 22 | Report all analytic inputs (such as values, ranges, references) including uncertainty or distributional assumptions. | Results, Table 1 |
| **Summary of main results** | 23 | Report the mean values for the main categories of costs and outcomes of interest and summarise them in the most appropriate overall measure. | Results, Table 2 and Table 3 |
| **Effect of uncertainty** | 24 | Describe how uncertainty about analytic judgments, inputs, or projections affect findings. Report the effect of choice of discount rate and time horizon, if applicable. | Results, Figure 1 and 2 |
| **Effect of engagement with patients and others affected by the study** | 25 | Report on any difference patient/service recipient, general public, community, or stakeholder involvement made to the approach or findings of the study | Not applicable |
| **Discussion** |  |  |  |
| **Study findings, limitations, generalisability, and current knowledge** | 26 | Report key findings, limitations, ethical or equity considerations not captured, and how these could affect patients, policy, or practice. | Discussion |
| **Other relevant information** |  |  |  |
| **Source of funding** | 27 | Describe how the study was funded and any role of the funder in the identification, design, conduct, and reporting of the analysis | Funding |
| **Conflicts of interest** | 28 | Report authors conflicts of interest according to journal or International Committee of Medical Journal Editors requirements. | COI |
